# Neurophysiological correlates of memory change in children with fetal alcohol spectrum disorders treated with choline

**DOI:** 10.3389/fpsyg.2022.936019

**Published:** 2022-09-26

**Authors:** Anita J. Fuglestad, Neely C. Miller, Birgit A. Fink, Christopher J. Boys, Judith K. Eckerle, Michael K. Georgieff, Jeffrey R. Wozniak

**Affiliations:** ^1^Department of Psychology, University of North Florida, Jacksonville, FL, United States; ^2^Masonic Institute for the Developing Brain, University of Minnesota Twin Cities, Minneapolis, MN, United States; ^3^Department of Psychiatry & Behavioral Sciences, University of Minnesota Twin Cities, Minneapolis, MN, United States; ^4^Department of Pediatrics, University of Minnesota, Minneapolis, MN, United States

**Keywords:** fetal alcohol spectrum disorders (FAS; FASD), choline, clinical trial, memory, event-related potentials, treatment, hippocampus

## Abstract

**Background:**

Prenatal and early postnatal choline supplementation reduces cognitive and behavioral deficits in animal models of Fetal Alcohol Spectrum Disorder (FASD). In a previously published 9-month clinical trial of choline supplementation in children with FASD, we reported that postnatal choline was associated with improved performance on a hippocampal-dependent recognition memory task. The current paper describes the neurophysiological correlates of that memory performance for trial completers.

**Methods:**

Children with FASD (*N* = 24) who were enrolled in a clinical trial of choline supplementation were followed for 9 months. Delayed recall on a 9-step elicited imitation task (EI) served as the behavioral measure of recognition memory. Neurophysiological correlates of memory were assessed *via* event-related potentials (ERP).

**Results:**

Delayed recall on EI was correlated with two ERP components commonly associated with recognition memory in young children: middle latency negative component (Nc amplitude; range: *r* = −0.41 to *r* = −0.44) and positive slow wave (PSW area under the curve; range: *r* = −0.45 to *r* = −0.63). No significant ERP differences were observed between the choline and placebo groups at the conclusion of the trial.

**Conclusion:**

Although the small sample size limits the ability to draw clear conclusions about the treatment effect of choline on ERP, the results suggest a relationship between memory performance and underlying neurophysiological status in FASD. This trial was registered.^1^

## Introduction

Children with Fetal Alcohol Spectrum Disorder (FASD) present with a broad range of neurocognitive deficits, from global intellectual impairments to specific cognitive deficits, including short-term and long-term memory deficits (Mattson et al., 2011). The hippocampus, and the recognition memory processes dependent on it, are consistently impacted by prenatal alcohol exposure (PAE) in animal models (Berman and Hannigan, 2000; Livy et al., 2003; Mattson et al., 2011). PAE has also been found to be associated with the structure and function of the hippocampus and its connectivity to brain regions, such as the prefrontal cortex, involved in memory and learning in children (Roediger et al., 2021).

Supplementation with the essential nutrient choline during fetal and early development reduces the severity of the learning and memory deficits caused by PAE in animal models (Thomas et al., 2000, 2007). Consistent with these preclinical findings, choline supplementation has been shown to improve memory functioning in young children with FASD immediately following choline supplementation (Wozniak et al., 2015) and 4 years-later (Wozniak et al., 2020). During a clinical trial of choline supplementation in children ages 2–5 years with FASD, choline improved hippocampus-mediated memory performance in the youngest children (2.5 to ≤4.0 years) immediately following the supplementation period, but not in the older children (4.0 to 5 years; Wozniak et al., 2015). As part of this original clinical trial of choline supplementation, we also assessed the neurophysiological underpinnings of these observed improvements in behavioral memory performance immediately following the period of choline supplementation, which we report here.

In young children, event-related potentials (ERP) are uniquely suited to investigating the underlying neural basis of memory (Deboer et al., 2007). Scalp electrodes record brain electrical activity from regional populations of neurons in response to specific stimuli. Two components of the recorded waveform are associated with recognition memory: the middle latency negative component (Nc) and the positive slow-wave component (PSW; Bauer et al., 2003, 2006; Riggins et al., 2009b). The Nc consists of a negative peak between approximately 400-800 ms post-stimulus and is prominent over frontal-central regions. It is generally thought that the Nc originates from areas of the prefrontal cortex and anterior cingulate cortex and reflects the saliency of a stimulus, with greater attention to a stimulus producing a larger Nc response. This response is affected by memory, with familiar and novel stimuli eliciting differential Nc amplitudes (Reynolds and Richards, 2005; De Haan, 2007). The PSW appears 750-1,500 ms after stimulus presentation and is distributed more widely across the scalp. This component is thought to reflect the process of encoding a stimulus and memory for contextual details (Nelson, 1994; De Haan, 2007; Riggins et al., 2013). PSW activity is thought to originate from temporal cortical areas and differs according to stimulus novelty (Nelson, 1994; De Haan, 2007; Riggins et al., 2013); however, the direction of the effect (greater PSW response to familiar stimuli vs. greater PSW response to novel) varies across studies.

One aim of the current ERP analysis was to assess for potential choline vs. placebo treatment effects at the level of neurophysiology. These results were primarily non-significant, possibly because of the small sample size, and are presented here only briefly. A second aim was to determine the relationship between performance on the elicited imitation (EI) behavioral memory task and underlying neurophysiological indicators of recognition memory to fully characterize the behavioral changes observed in the published trial results. We hypothesized that EI memory performance would be associated with distinct ERP components (Nc and PSW). Specifically, we hypothesized that EI performance would be correlated with Nc activity in anterior regions and with PSW activity across scalp regions. Hypotheses about the direction of the correlations were not made *a priori* as the literature has shown that familiar and unfamiliar stimuli can elicit differential magnitudes of Nc and PSW responses across development and in response to varying task demands (e.g., Bauer et al., 2006).

## Materials and methods

### Participants

Children with FASD (*n* = 40), ages 2.5 to 5 years at enrollment were recruited from an FASD Clinic and an Adoption Medicine Clinic and enrolled in a 9-month randomized, double-blind, placebo-controlled trial of choline (ClinicalTrials.Gov #NCT01149538). All procedures were approved by a University IRB, and all participants underwent an informed consent process.

The trial has been previously described in detail (Wozniak et al., 2013, 2015). Enrollment required meeting modified Institute of Medicine (IOM) criteria for FASD (Hoyme et al., 2005). Among the participants in the second wave of the trial (Wozniak et al., 2015), 19 participants (placebo: *n* = 9; choline: *n* = 10) had usable ERP data at *both* study baseline and study completion. Only participants with data at both baseline and study completion were included in the between-group analyses examining the differences in the ERP response as a function of treatment (aim 1). Data were missing from participants who did not undergo the ERP procedures (*n* = 4), did not contribute enough artifact-free trials (baseline assessment: *n* = 5; 9-month assessment: *n* = 6), or discontinued trial participation (*n* = 6). Correlations between ERP and EI memory performance (aim 2) were examined at study completion (9-month assessment) and included all participants who had usable ERP data at study completion, regardless of their baseline data (*n* = 24). Five of the 24 participants had usable ERP data at study completion without usable baseline data. Of those participants who completed the full 9-month trial, 71% (24 of 34) had usable ERP data at study completion which is comparable to, or slightly higher than, similar ERP studies in children with PAE (e.g., Burden et al., 2011).

Sample characteristics (*N* = 24) are described in Table 1. The participants with usable ERP data at study completion included in the analyses were slightly older, scored higher on the Mullen Scales of Early Learning, and had milder forms of FASD (partial FAS or ARND vs. FAS) than those without usable data. Participants with and without usable ERP data differed by race (a significant number of children who were white had unusable ERP data, and Native American children had more usable data). The reason for this difference was not apparent from the data.

**Table 1:** Characteristics at study enrollment of the subsample with usable ERP data.

N(%) or mean (SD)	Participants with ERP data at completion (9-month assessment) (*n* = 24)	Participants without ERP data at completion (9-month assessment) (*n* = 16)	
Age at enrollment (years)	3.97 (0.85)	3.43 (0.69)	*t*(38) = −2.14, *p* = 0.039
**Gender**			
Male	7 (29%)	8 (50%)	*p* = 0.205
Female	17 (71%)	8 (50%)	
**Racial Categories**			
White	4 (17%)	10 (63%)	*p* = 0.007
Black or African American	6 (25%)	4 (25%)	
American Indian/Alaska Native	7 (29%)	0 (0%)	
Asian	1 (4%)	1 (6%)	
More than one race	6 (25%)	1 (6%)	
**Ethnic Category**			
Hispanic or Latino	0 (0%)	0 (0%)	*p* = 0.508
Not Hispanic or Latino	22 (92%)	16 (100%)	
Unknown	2 (8%)	0 (0%)	
**Dysmorphic Facial Features**			
Lip (score 4 or 5)	12 (50%)	8 (50%)	*p* = 1.000
Philtrum (score 4 or 5)	10 (42%)	6 (38%)	*p* = 1.000
Palpebral Fissure (≤10^th^ percentile) ^1^	18 (75%)	12 (75%)	*p* = 1.000
≥ 2 Facial Features Present	13 (54%)	8 (50%)	*p* = 1.000
**Growth Deficiency (≤10**^th^ **percentile)**			
Height ^1^	1 (4%)	1 (6%)	*p* = 1.000
Weight	1 (4%)	1 (6%)	*p* = 1.000
**Deficient Brain Growth (≤10**^th^** percentile)**			
Occipital-Frontal Circumference (OFC)	0 (0%)	1 (6%)	*p* = 0.400
**Alcohol Exposure**			
Alcohol Confirmed	18 (75%)	15 (94%)	*p* = 0.210
Alcohol Suspected	6 (25%)	1 (6%)	
**Drug Exposure**			
Other Drug Exposure Suspected	20 (83%)	9 (56%)	*p* = 0.80
**IOM Diagnostic Category** ^1^			
FAS	0 (0%)	4 (25%)	*p* = 0.041
Partial FAS	14 (58%)	7 (44%)	
ARND	10 (42%)	5 (31%)	
**Treatment Group**			
Choline	11 (46%)	10 (62%)	*p* = 0.289
Placebo	13 (54%)	6 (38%)	
**Baseline Cognitive Functioning** ^2^			
Mullen Visual Reception	43 (12)	33 (11)	*t*(36) = −2.53, *p* = 0.016
Mullen Fine Motor	40 (12)	37 (13)	*t*(36) = −0.77, *p* = 0.445
Mullen Receptive Language	41 (11)	32 (8)	*t*(36) = −2.49, *p* = 0.018
Mullen Expressive Language	43 (9)	36 (9)	*t*(36) = −2.52, *p* = 0.016
Mullen Early Learning Composite	85 (17)	72 (13)	*t*(36) = −2.48, *p* = 0.018

Comparisons between treatment groups were conducted using Independent Samples t-tests for continuous variables and Fisher’s Exact Test for categorical variables.

1 FAS: Fetal Alcohol Syndrome; ARND: Alcohol-Related Neurodevelopmental Disorder.

2 n = 2 in the group without ERP data did not have Mullen data at enrollment.

### Study design

Complete descriptions of the 9-month clinical trial methods and procedures are available in Wozniak et al. (2015). Participants were randomly assigned in a one-to-one allocation to receive 500 mg choline (1.25 g choline bitartrate) or a placebo daily for 9 months. The allocated intervention was a powdered fruit-flavored drink mix that was developed for the study. Parents were instructed to administer 1 dose/day by mixing it with 4 fl oz. (118.3 ml) H2O. The research team and participants were blinded to group assignments.

Cognitive (global cognitive and hippocampal-dependent memory) and ERP assessments occurred at baseline (prior to receiving choline or placebo), at 6-months, and at 9-months (study completion). Results for global cognitive functioning (primary outcome) and hippocampal-dependent memory (secondary outcome) have been reported previously (Wozniak et al., 2015). ERP, a secondary outcome measure in the trial, is the focus of the current analyses.

EI assessments occurred at each visit (baseline, 6-months, and study-completion). EI was administered prior to the ERP assessment. Only ERP data from the baseline and the concluding visit (9-months) are reported here. These two time points were included in analyses to best capture the pre-and post-treatment changes in participants with complete data (aim 1) and to limit the number of correlational analyses (aim 2).

### Elicited imitation

EI assesses long-term memory in young children through behavioral imitation of action sequences (Bauer, 1995) and requires support from the hippocampus (Mcdonough et al., 1995). In this study, each event sequence was themed (e.g., going camping) and included nine individual actions with multiple toys that were presented in a prescribed order (see Wozniak et al., 2015 for complete methods).

During the task, an experimenter modeled nine-item event sequences involving a set of themed toys twice with narration. For instance, during the event sequence called “going camping,” the experimenter modeled nine individual items, such as baiting a hook, catching a fish, roasting a marshmallow, and setting up a tent, in the prescribed order. After the experimenter modeled the nine-item sequence twice, the child was directed to use the toys to repeat the event sequence either immediately (immediate condition) or following a 15-min delay (delay condition). Three event sequences were administered to each participant: one event sequence for the immediate condition and two event sequences for the delay condition. Only data from the delay condition are included in the current analysis as a measure of hippocampal-dependent long-term memory.

Variables used in analyses included the percentage of correct individual event items (e.g., baiting a hook, catching a fish; maximum = 9 items) and the percentage of correctly-ordered item pairs (e.g., baiting the hook before catching the fish; maximum = 8 ordered pairs) following the delay, averaged across the two delay event sequences. Sessions were video-recorded and scored off-line by trained raters. Twenty percent of videos were coded by multiple raters to ensure reliability (93%).

Age was correlated with recall of individual event items, *r = 0*.46, *p =* 0.019, but not with recall of ordered item pairs, *r = 0*.29, *p =* 0.147. To control for the effect of age on memory performance, all EI data were adjusted for age. Standardized residuals, saved from regressing EI performance on age, were used in analyses.

### Event-related potentials

#### ERP collection and processing

After obtaining head circumference measurements, children were fitted with a 128-channel Geodesic Sensor Net (Electrical Geodesics, Inc). Scalp impedances were measured using NetStation software and adjusted until below 50 KΩ. EEG data were collected and recorded using NetAmps Amplifiers (EGI, Inc., Eugene, OR) and referenced to a single vertex electrode (sampling rate = 250 Hz; filter = 0.1-100 Hz bandpass; gain = 10,000x) using NetStation software. A 30-Hz low-pass filter was applied offline and data were segmented and baseline-corrected to the average voltage during the 100 ms pre-stimulus onset. Data were visually inspected for motion or electroocculogram (EOG) artifact; trials containing more than 12 bad channels were rejected (see Deboer et al., 2007 for procedures used in developmental populations), and spherical spline interpolation was used to replace individual bad channels on remaining trials. Individual participant averages were calculated for each condition (familiar or unfamiliar; see “ERP Stimuli” for description) and re-referenced to the average reference.

Component time windows were chosen based on previous literature and visual inspection. For Nc, adaptive mean amplitude and latency to peak amplitude were derived from a 350-700 ms window; for PSW, average area values were derived from a 750-1,500 ms window.

**Figure 1:**
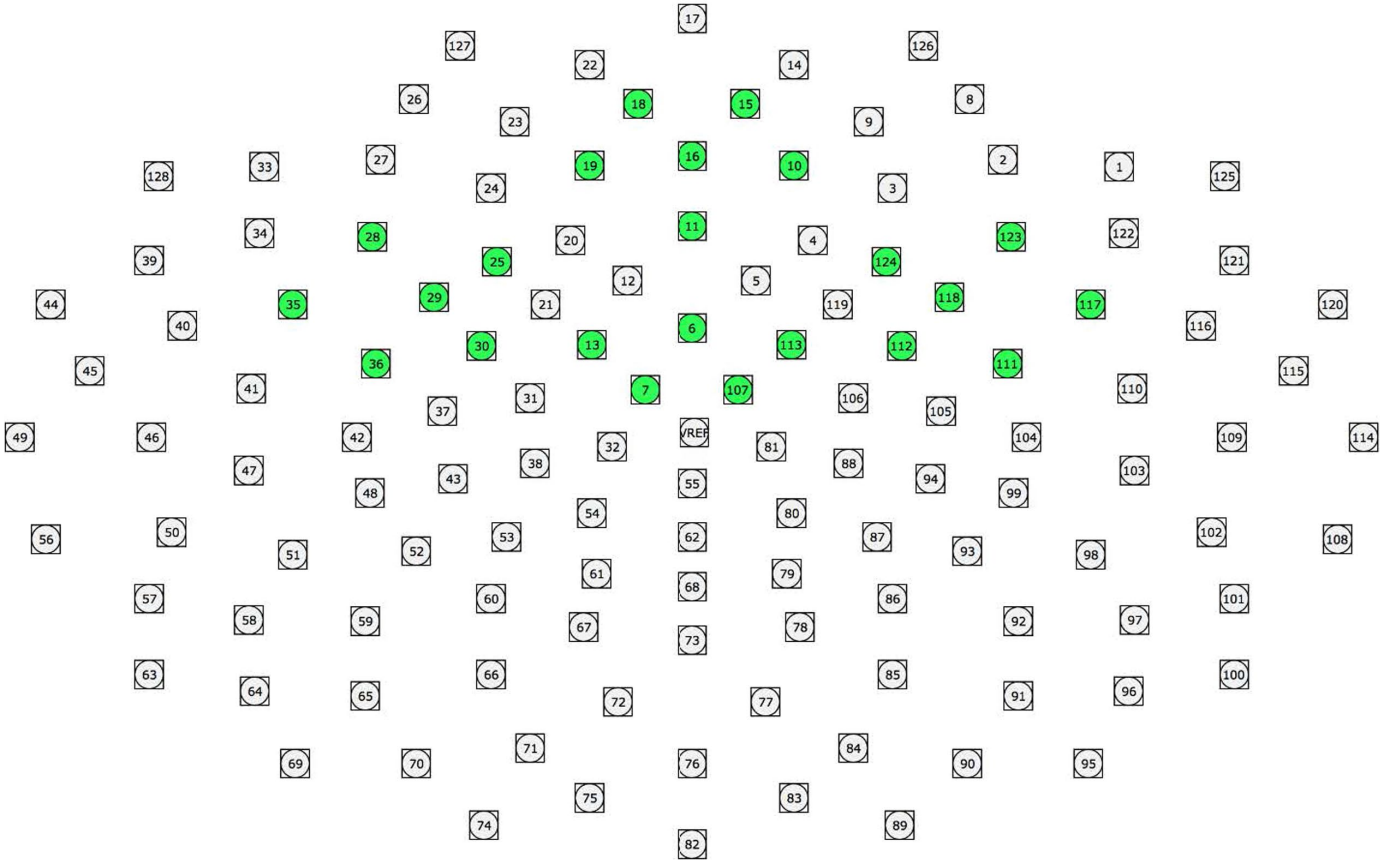
ERP clusters used in data analyses. Clusters were created to represent the electrophysiological response to stimuli in different scalp regions. ERP data collected from the individual sensors were averaged together to represent the electrophysiological activity at the following scalp locations: Prefrontal (18, 15, 19, 16, 10, 11), Right Frontal (123, 124, 118, 112, 111, 117), Left Frontal (28, 25, 29, 30, 36, 35), and Central (13, 6, 113, 7, 107).

Clusters were created to represent the electrophysiological response to stimuli in different scalp regions. ERP data collected from the individual sensors were averaged together to represent the electrophysiological activity at the following scalp locations: Prefrontal (18, 15, 19, 16, 10, 11), Right Frontal (123, 124, 118, 112, 111, 117), Left Frontal (28, 25, 29, 30, 36, 35), and Central (13, 6, 113, 7, 107; Figure 1).

#### ERP stimuli

During ERP recording, participants were shown pictures of toys used in the preceding EI session (familiar) and pictures of novel toys (unfamiliar). Each picture displayed one of the individual steps of an EI event sequence. A single picture of the completed event sequence was also presented, resulting in 10 pictures per EI event sequence. A total of 120 stimuli were shown to each participant; each picture was presented randomly six times (60 pictures per condition). Familiarity response scores were calculated by subtracting the ERP response to unfamiliar stimuli from the familiar stimuli response for both Nc and PSW components at each region of interest.

## Results

### Treatment group differences in the ERP familiarity response

Independent samples *t-*tests were used to test for a treatment effect on change in the ERP familiarity response scores between baseline and completion (9-month assessment). There was no significant difference between choline and placebo groups in change of the ERP familiarity response over the 9 months (Table 2).

**Table 2:** Change in the ERP familiarity response (familiar-unfamiliar) from baseline to the 9-month follow-up as a function of treatment.

	Placebo *n* = 9	Choline *n* = 10	
	*M* (*SD*)	*M* (*SD*)	*t* test
**Nc Adaptive Mean Amplitude (μV)**			
Prefrontal	−4.02 (9.07)	3.36 (10.56)	*p* = 0.123
Right frontal	−0.47 (3.25)	−0.39 (5.97)	*p* = 0.972
Left frontal	−0.01 (7.06)	1.14 (5.43)	*p* = 0.695
Central	−3.17 (5.36)	2.06 (6.12)	*p* = 0.065
**Nc Latency Peak Amplitude (ms)**			
Prefrontal	53.06 (94.89)	−44.08 (113.51)	*p* = 0.061
Right frontal	8.46 (64.62)	3.18 (117.02)	*p* = 0.906
Left frontal	44.04 (79.61)	−29.45 (93.73)	*p* = 0.085
Central	34.64 (85.44)	−41.42 (98.68)	*p* = 0.092
**PSW (area under the curve)**			
Prefrontal	−4.19 (8.77)	1.89 (13.89)	*p* = 0.276
Right frontal	0.93 (3.03)	0.42 (5.68)	*p* = 0.812
Left frontal	0.02 (6.12)	1.31 (4.58)	*p* = 0.607
Central	−2.36 (5.35)	2.60 (8.01)	*p* = 0.135

These analyses include participants who had usable ERP data at both baseline and the 9-month follow-up (*n* = 19). Of the 24 participants with usable ERP data at follow-up, five had missing ERP data at baseline due to too few artifact-free trials.

### Correlations between the ERP familiarity response and elicited imitation performance

Correlations were examined between the two EI variables (individual event items and ordered item pairs) standardized for age and the familiarity ERP response for each component (Nc and PSW) at each of the four scalp regions outlined above. To limit the number of analyses, only data from the 9-month visit (study completion) were utilized for these analyses.

#### Nc component

Nc adaptive mean amplitude was correlated with EI recall of individual event items and ordered item pairs at prefrontal and right frontal regions (range: *r* = −0.411 to *r* = −0.439; Table 3). All Nc mean amplitude and EI correlations were negative. That is, better delayed recall performance during the EI tasks was associated with greater Nc amplitude to familiar stimuli compared to unfamiliar stimuli.

**Table 3:** Correlations between elicited imitation memory performance and ERP familiarity response (familiar-unfamiliar) at the end of the 9-month intervention (*n* = 24).

	Recall of Individual Items	Recall of Ordered Pairs
**Nc Adaptive Mean Amplitude**		
Prefrontal	−0.439^*^	−0.373^†^
Right frontal	−0.417^*^	−0.411^*^
Left frontal	−0.140	0.001
Central	−0.319	−0.339
**Nc Latency to Peak Amplitude**		
Prefrontal	0.005	0.191
Right frontal	0.043	0.105
Left frontal	−0.394^†^	−0.371^†^
Central	−0.294	−0.299
**PSW (area under the curve)**		
Prefrontal	−0.625^**^	−0.609^**^
Right frontal	−0.032	−0.094
Left frontal	−0.453^*^	−0.134
Central	−0.455^*^	−0.290

Elicited imitation memory performance is standardized for age. Interpretation of a negative correlation means that better delayed recall is associated with a greater Nc amplitude for familiar stimuli (compared to unfamiliar), a shorter Nc latency to familiar stimuli (compared to unfamiliar), and a greater PSW response to unfamiliar stimuli (compared to familiar).

† *p *< 0.10;

* *p *< 0.05;

** *p *< 0.01.

Only marginally significant correlations were found between EI and Nc latency at the left frontal location (range: *r =* −0.371 to *r =* −0.394; Table 3). Better delayed recall tended toward shorter Nc latency to familiar stimuli compared to unfamiliar.

#### PSW component

EI performance on individual event items and ordered item pairs was negatively correlated with PSW. Individual event item recall was correlated with PSW at prefrontal, left frontal, and central scalp locations (range from *r =* −0.453 to *r =* −0.625; Table 3), whereas ordered item pair recall was correlated only at the prefrontal scalp location. All correlations were negative, meaning that better EI recall was associated with greater PSW response to unfamiliar stimuli compared to familiar stimluli.

## Discussion

These results indicate that behavioral performance on the EI memory paradigm is associated with underlying memory-related neurophysiological responses in FASD. A statistically significant treatment effect for choline was not seen for the ERP measures of memory.

The lack of treatment effect may be from limited power to detect any between-group differences in the ERP familiarity response – due to both the variability in the ERP data and to the small number of participants in each group after removing participants with incomplete or unusable data. It is also possible that the overall treatment effect was diluted by the slightly older age of the participants with usable ERP data. Several participants with the largest treatment response in behavioral memory performance were excluded from the current analyses due to incomplete ERP data. The initial treatment trial demonstrated that choline improves recognition memory performance in younger children to a greater extent than older children at study completion (Wozniak et al., 2015). That finding is consistent with the preclinical literature demonstrating that choline improves hippocampal function in rodents exposed to alcohol prenatally, especially when choline is given early in development. The hippocampus has its most rapid period of growth and differentiation in humans from 28 weeks gestation to approximately 18 months postnatal age. Therefore, interventions aimed at improving hippocampal development are likely to be more efficacious at younger ages.

Nonetheless, the ERP familiarity response was associated with behavioral recall on the EI task. Better recall was correlated with a larger Nc response to familiar items in anterior regions and with a greater PSW response to unfamiliar items. These correlations are consistent with ERP responses on observed in other young populations (e.g., Riggins et al., 2009a) and provide evidence for the validity of the EI task as a measure of hippocampally-mediated memory functioning in young children with FASD. This is particularly important for future studies in this population because performance on the behavioral memory task may be influenced by factors other than memory ability. For instance, challenging behaviors that are common in children with FASD, such as inattention, hyperactivity, poor cooperation, and/or self-regulation difficulties (Mattson et al., 2011), could reduce the accuracy or validity of behavioral measures of memory.

Associations between memory and the ERP response suggest that the memory improvements observed in this sample may be due to underlying neurophysiological changes associated with choline supplementation. Potential neural mechanisms for choline’s effects on memory during development include the production of cell membrane phospholipids for axonal growth and myelination, enhancement of acetylcholine, and epigenetic effects related to DNA methylation. In animal models of FASD, choline supplementation affects the hippocampal cholinergic system (Monk et al., 2012) and alters brain structure and function in regions essential for memory functioning, including methylation in the hippocampus and prefrontal cortex (Otero et al., 2012). It is worth noting that prenatal choline supplementation may alter DNA methylation (Otero et al., 2012), but the effects of postnatal choline in 2–4 year old children are more likely related to acetylcholine alterations and/or effects on the developing white matter.

## Conclusion

FASD is a highly prevalent condition, affecting 2–5% of children in the United States (May et al., 2018) and thus far, there are very few behavioral, cognitive and/or biological interventions specific for FASD (Kable et al., 2007; Bertrand, 2009; Nash et al., 2014). Although ERP did not prove to be statistically sensitive to choline treatment in this small trial, the associations between memory performance and neurophysiological functioning are important in understanding the context of the effects of choline supplementation on memory functioning in young children with FASD. The effects of choline in the sample described here were even more apparent 4 years after choline supplementation, with improvements in verbal memory, working memory, non-verbal intelligence, and visual–spatial skills compared to the placebo group (Wozniak et al., 2020). Another recent human study of choline supplementation (prenatal, in this case) has demonstrated treatment effects in FASD at the neurophysiological level (eye blink conditioning response) as well as the cognitive and growth levels (Jacobson et al., 2018). This accumulating evidence suggests that choline warrants further study as a biological treatment to improve outcomes in FASD and that multiple levels of assessment – including neurophysiologic – are important in fully characterizing these effects.

## Data availability statement

The datasets presented in this article are not readily available because they are part of an ongoing longitudinal study (R01AA024123; NCT05108974). Requests to access the datasets should be directed to Jeffrey Wozniak, Ph.D, jwozniak@umn.edu.

## Ethics statement

The studies involving human participants were reviewed and approved by University of Minnesota IRB. Written informed consent to participate in this study was provided by the participants' legal guardian/next of kin.

## Author contributions

Each author has participated in the concept and design of the study, the collection and analysis of the data, and the preparation/review of the manuscript. Each author acknowledges that care has been exercised in the preparation of the data. All authors contributed to the article and approved the submitted version.

## Funding

This work was supported by the National Institute on Alcohol Abuse and Alcoholism (5R21AA019580, R33AA019580 and R01AA024123).

## Conflict of interest

The authors declare that the research was conducted in the absence of any commercial or financial relationships that could be construed as a potential conflict of interest.

## Publisher’s note

All claims expressed in this article are solely those of the authors and do not necessarily represent those of their affiliated organizations, or those of the publisher, the editors and the reviewers. Any product that may be evaluated in this article, or claim that may be made by its manufacturer, is not guaranteed or endorsed by the publisher.
